# Measuring Dental Enamel Thickness: Morphological and Functional Relevance of Topographic Mapping

**DOI:** 10.3390/jimaging9070127

**Published:** 2023-06-23

**Authors:** Armen V. Gaboutchian, Vladimir A. Knyaz, Evgeniy N. Maschenko, Le Xuan Dac, Anatoly A. Maksimov, Anton V. Emelyanov, Dmitry V. Korost, Nikita V. Stepanov

**Affiliations:** 1 Medical Institute, Peoples’ Friendship University (RUDN), 117198 Moscow, Russia; 2 Phystech School of Applied Mathematics and Informatics, Moscow Institute of Physics and Technology (MIPT), 141701 Dolgoprudny, Russia; 3 State Research Institute of Aviation Systems (GosNIIAS), 125319 Moscow, Russia; 4 Borissiak Paleontological Institute, Russian Academy of Sciences, 117647 Moscow, Russia; 5 Joint Russian-Vietnamese Tropical Scientific and Technological Center, Hanoi 650000, Vietnam; 6 Institute of Tropical Ecology of the Joint Russian-Vietnamese Tropical Scientific and Technological Center, Hanoi 650000, Vietnam; 7 Faculty of Geology, Moscow State University, 119991 Moscow, Russia

**Keywords:** enamel thickness, dental morphology, dental occlusion, dental enamel, topographic mapping, micro-focus computed tomography, palaeontology, *Gigantopithecus*, orangutan

## Abstract

The interest in the development of dental enamel thickness measurement techniques is connected to the importance of metric data in taxonomic assessments and evolutionary research as well as in other directions of dental studies. At the same time, advances in non-destructive imaging techniques and the application of scanning methods, such as micro-focus-computed X-ray tomography, has enabled researchers to study the internal morpho-histological layers of teeth with a greater degree of accuracy and detail. These tendencies have contributed to changes in established views in different areas of dental research, ranging from the interpretation of morphology to metric assessments. In fact, a significant amount of data have been obtained using traditional metric techniques, which now should be critically reassessed using current technologies and methodologies. Hence, we propose new approaches for measuring dental enamel thickness using palaeontological material from the territories of northern Vietnam by means of automated and manually operated techniques. We also discuss method improvements, taking into account their relevance for dental morphology and occlusion. As we have shown, our approaches demonstrate the potential to form closer links between the metric data and dental morphology and provide the possibility for objective and replicable studies on dental enamel thickness through the application of automated techniques. These features are likely to be effective in more profound taxonomic research and for the development of metric and analytical systems. Our technique provides scope for its targeted application in clinical methods, which could help to reveal functional changes in the masticatory system. However, this will likely require improvements in clinically applicable imaging techniques.

## 1. Introduction

Teeth constitute one of the main types of objects studied in palaeontology, physical anthropology, and other disciplines. This is due to their highly mineralised tissue composition that increases their preservation or fossilisation potential over skeletal bones. For this reason, teeth have been studied using diverse techniques to provide information that has increased our understanding of various aspects in natural sciences [1,2]. However, as many issues remain unresolved and require further research, we focus on the methodological problems in enamel thickness measurement techniques.

Studies on dental enamel thickness go back more than a hundred years. During the early decades of study, the focus was mainly on naturally fractured teeth. Those studies showed that the thickness of dental enamel differs in various species (in this regard, the works of G.E. Pilgrim are often quoted [3]). However, one of the main problems was that randomly inclined surfaces of naturally fractured teeth could not provide reliable data for consistent comparisons. In order to obtain a sufficient level of measurement accuracy, new techniques were developed [4], leading to the emergence of destructive methods. Thus began the period of sectioning teeth (e.g., Molnar and Gantt [5]), and from that moment, researchers have worked on improving the precise positioning of teeth and instruments used to obtain accurate sections.

With the established approach, teeth were cut in the bucco–lingual direction through the dentine horn tips (although first attempts made using modern techniques have shown that those approaches of cutting through the targeted morphological structures were imprecise [6]). However, using the above-mentioned destructive techniques provided visualisation of the contours in uniformly orientated sections of dental tissues that could then be studied in greater detail (at least when compared with naturally fractured material). Therefore, planar enamel thickness measurement techniques were rapidly developed both in terms of systematising constructions and introducing parameters based on dental ontogeny and morphological interpretation. It was thus proposed that the average enamel thickness parameter should be used for basic metric assessment. To calculate the average enamel thickness parameter the enamel area in the section (or enamel cap volume), should be divided by the enamel–dentine junction contour length (or area) [7]. Modification of this parameter is also used in the current study. It is worth mentioning that destructive enamel thickness measurement techniques are still being used, for instance when combined with daily enamel secretion rate studies [8].

Nevertheless, the search for new study techniques has never ceased, spurred on by the most significant drawback in the sectioning technique, the inevitable loss of material (at least equal to the saw thickness). In addition, the separation of teeth into pieces, especially when the studied items are unique, may be an unwelcome approach (unless histological aspects become a part of the research). It should be noted that a number of alternative study techniques have been applied to measure enamel thickness. Some of them have used non-destructive approaches, such as studies of enamel around exposed dentine areas on worn-out teeth [9]. However, such approaches can be grouped with studies of naturally fractured teeth in terms of the unpredictability of the obliquity angle of the exposed enamel plane. Other methods have suggested even more destructive tactics than sectioning, such as removing the outer enamel layer of the studied teeth [10]. Nevertheless such methods, despite their technical complexity, could provide a decent picture of the inner dental morphology and enamel thickness, especially when combined with scanning techniques [11].

It is no exaggeration to say that imaging has contributed substantially to the development of modern methods for enamel thickness measurements. Insofar as these study techniques require the depiction of the inner layers of teeth, X-ray tomographic scanning is widely used today. In some cases though, neutron imaging can be applied depending on the physical properties of the material studied [12,13]. Another research component is when enamel thickness measurements are carried out in parallel with enamel structure analyses on a histological level; synchrotron micro-tomographic scanners should be used for such studies [14,15]. Nevertheless, X-ray tomography had been criticised for the wide use of sectioning techniques. Thus, when data obtained through sectioning are taken as reference, researchers report discrepancies of up to 50% compared with conventional radiographs [7,16]).

It is certainly possible to understand the effect of superimposition from tooth undulations on a planar X-ray image; however, it is interesting to note that later assessments [6] showed a possibility of overestimation in enamel thickness values measured in sections (in the range of 20–90% at the cusp tips). There have also been reports of the low-resolution potential of conventional radiographic images at the border of the enamel and dentine on fossilised teeth [7,17]. These data are partially consistent with earlier attempts of applying computed tomographic scanning in enamel thickness research, which cannot be considered as successful. Technical problems with the beam hardening effect and the relatively low spacial resolution of tomographic scans compared with the sizes of the studied morphological structures did not permit many researchers to have a preference for X-ray imaging applications in enamel thickness studies [18]. Nevertheless, the non-destructiveness of tomographic scanning was an important enough factor for the further application of the method in research [19], especially since the majority of the above-mentioned issues related to X-ray techniques have been successfully resolved in micro-focus computed tomography.

Image processing and analysis techniques are just as important as the imaging methods that contribute to enamel thickness studies. There are no digital 3D reconstructions without image processing which offers very basic conventional benefits of using 3D models (e.g. marking points and sectioning multiple times, sharing study results with colleages, re-measuring, etc.). Even though the latter are also important, there is a wide range of methodological modifications of recognised techniques that can be easily applied to tests avoiding any damage to the study material [20]. However, with the range of approaches for topographic analyses [21,22], the possibility exists to develop automated orientation and measurement algorithms based on surface curvature and a point coordinate analysis [23,24]. This can be achieved using digital techniques and 3D reconstructions. The aforementioned important features are used today in a variety of studies, in evolutionary research, taxonomical assessments, and morphological studies [21,25,26], representing, in some cases, methodological extensions of techniques proposed for studies on real teeth [27]. The combination of enamel thickness measurements with other techniques, e.g., geometric morphometrics, is also characteristic for current odontological studies [28].

In line with the technological and exploratory advantages of the 3D digital techniques discussed above, we pay attention to some traditional approaches for understanding dental morphology used in enamel thickness measurement techniques, especially in sectioning real teeth or their digital reconstructions [7,29]. We note that it is the bucco–lingual section that forms the basis and runs throughout the majority of studies, effectively reflecting the most traditional concept corresponding to the basic morphology of posterior teeth. Indeed, any molar or premolar, be it upper or lower, right or left, regardless of the number of cusps, sectioning in the bucco–lingual direction reveals approximately the same pattern of two elevations, which makes the contour very recognisable. These views are based on concepts dating back to at least the late nineteenth century, as descriptions of the arrangement of teeth in terms of their overlapping are represented by two distinct parts: the buccal and lingual sides of posterior teeth [30]. Since then, numerous attempts have been made in classifying these parts according to their potential function: stamp and shearing cusps [31], functional and non-functional [32], supporting and guiding [33], etc. Furthermore, it is worth noting that these views are often reflected in studies on enamel thickness as well, for example, as part of the interpretation of measurement results [29]. We showed in our previous works the importance of other distinct structures, such as the occlusal surface [34,35,36], and the technique presented here is largely based on the occlusal surface contours defined on the internal and external surfaces of the dental enamel. Through the proposed automated digital technique, it becomes possible to collect more objective, ample, versatile, and morphologically relevant data that can refer to measuring both teeth and enamel thickness. In addition, these methods can provide the fast accumulation of a large amount of data and facilitate the mapping of surfaces and volumes of teeth for studying their topographic features.

Today, enamel thickness measurements include a wide variety of samples that can be clustered in different groups. These studies include deciduous and permanent teeth [37,38], canines or molars [39,40], upper and lower teeth [41,42], etc. We should also note that a wide variety of species, predominantly primates, have been studied in terms of enamel thickness measurements for the assessment of their taxonomical relations, understanding their evolutionary processes, and many other topics such as studies of sexual dimorphism [43], dietary preferences [29], or methodological aspects of imaging and measuring techniques [44]. Thus, we can observe a long history of development, methodological improvements, and experience in measuring enamel thickness in teeth, and this continues to play one of the key roles in the study of taxonomic relations in palaeontology and palaeoanthropology. Numerous studies have shown different patterns of enamel thickness in extinct species; with remarkable differences between modern *Homo sapiens* and Hominoidea, allowing the assessment of the intraspecific variability. Nevertheless, enamel thickness remains a topical issue for studies that is relevant to and widely used in odontological research. Different perspectives have been debated, from the comparison of data obtained through 2D and 3D measurements and enamel thickness distribution patterns to topographic mapping and statistical analyses of measured and calculated parameters [45].

In this respect, we propose a range of methodological approaches that contribute to create closer links between modern technological advantages and dental morphology and function [46]. This is achieved in the current study through the application of automated algorithms for orientation and measuring enamel thickness, as well as for measurements between similar morphological elements on two surfaces. Such strategies are planned to be applied in studies of samples collected during expeditions into northern Vietnam and are presented in this article on *Gigantopithecus* and orangutan teeth. It should be mentioned that, in general, studies on the enamel thickness of extinct primates illustrate specific features in different groups within the Hominidae family and their divergence. One of the issues that can be resolved through detailed enamel studies refers to the differences between extant species of orangutan (*Pongo*) inhabiting the islands of Borneo and Sumatra. In addition, relations between extant and extinct (continental) species of *Pongo* sp. can be clarified through studies of their enamel structure and thickness. The general evolutionary tendency in the Ponginae subfamily shows a decrease in enamel thickness from the Late Miocene to the Late Pleistocene. Modern orangutans occupy extreme positions according to this feature. However, other members of the subfamily, phylogenetic relatives of orangutans—*Gigantopitheci* that inhabited territories in south-eastern Asia for more than 3 million years—had no changes in their relatively thick enamel. Up to the present day, we do not possess reliable data on the changes in enamel thickness in Pleistocene orangutans and *Gigantopitheci*; therefore, further studies on enamel morphometric features are of special interest in the evolutionary research of these species.

## 2. Materials and Methods

Paleontological material presented in the current article was obtained during excavations carried out in solutional caves washed-out in the limestone and dolomite rock in northern Vietnam. The larger of the two teeth is from the main chamber of the Lang Trang cave (Thanh Hoa province). Its lower galleries are filled with sediments containing the bones and teeth of mammals from the Middle and Late Pleistocene periods. Among the variety of findings, there are the teeth of primates that have been classified as belonging to an extinct continental subspecies of orangutan (*Pongo* sp.) and to another primates, the largest known representative of the order—*Gigantopithecus blacki* [47].

The other tooth was found in bone breccia of red loam soil from the Tham Khai (Binh Gia District) cave. Together with similar samples, it was tentatively classified as belonging to a sub-species of orangutan (*Pongo pygmaeus kahlkei*) that once inhabited the territories of the modern Vietnamese province of Lang Son [48,49].

**Figure 1:**
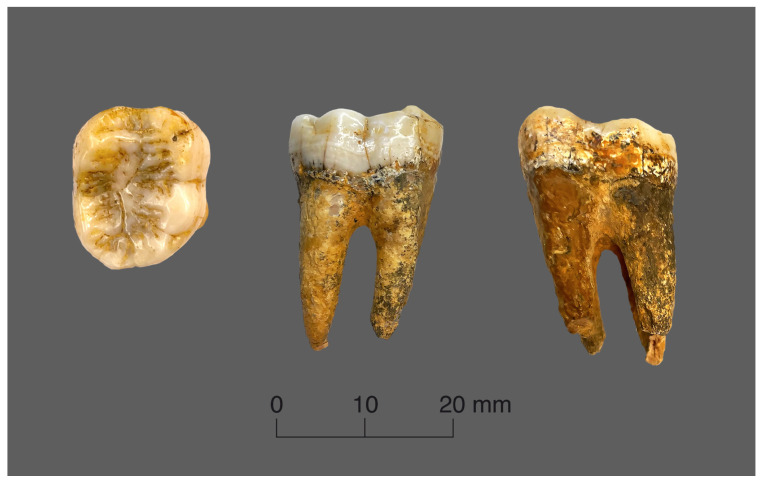
Views of the *Gigantopithecus* tooth.

The topographic mapping of the enamel thickness measurements was conducted for the lower right second molar of *Gigantopithecus* and the lower left second molar of orangutan. The *Gigantopithecus* tooth has completely preserved roots and coronal part (Figure 1). The principal morphological features are well-preserved, but the occlusal and mesial surfaces show some signs of natural wear with a small spot of exposed dentine on the mesial buccal cusp. The manually measured size of the tooth crown is 19.11 mm in the mesio–distal dimension and 16.62 mm in the bucco–lingual dimension, which correspond to the average size values of the primates’ lower molars.

**Figure 2:**
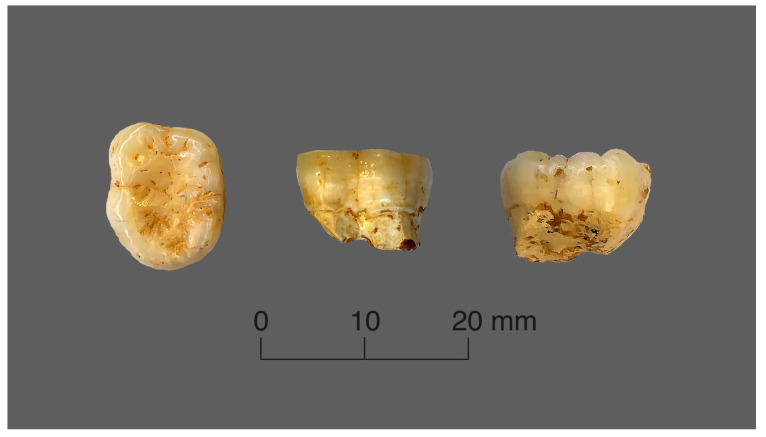
Views of the orangutan tooth.

The orangutan tooth has a completely preserved coronal part (except for it roots, which had apparently been eaten by porcupines) and a very similar wear degree, which was a decisive factor in choosing this sample (Figure 2).

The teeth were scanned using a Skyscan 1172 (Bruker, Germany) micro-focus computed tomographic scanner. The parameters for scanning the *Gigantopithecus* molar were the following: source voltage: 100 kV; source current: 100 μA; image pixel edge size: 12.55 μA; exposure: 2400 ms; rotation step: 1 deg. Three-dimensional models were generated after the raw data processing stage, which included image segmentation with the subsequent cleaning of the irrelevant areas (performed on Aviso 9.0 software). Separate models for the enamel and dentine layers were obtained; model surfaces were smoothed, and the size reduction and format change were performed. In addition to the typical preparation process of model generation, the enamel cap and dentine volumetric reconstructions underwent subsequent trimming, re-formatting (from .stl to .x), and converting the measurement unit (from micrometers (μm) to millimetres (mm)).

At the end of the CT image processing stage, the enamel cap models of the teeth were presented as a triangulated irregular net (TIN) that included both external and internal enamel surfaces. Therefore, in order to create a topographic map of the enamel thickness distribution, the TIN segmentation of the external and internal surfaces was performed.

The first step in this process is to translate the coordinate system origin to the centre of mass in the TIN model. Then, segmentation is performed using an analysis of the TIN point position and its normal direction according to Algorithm 1.
**Algorithm 1:** Tooth enamel cap segmentation
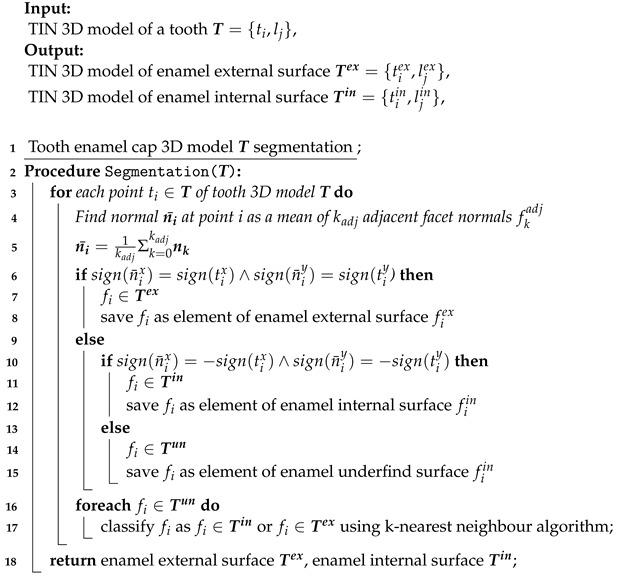


These procedures were performed using original software “Automated teeth 3D measurements—O3DO” [50] that was developed by the authors for the purpose of odontometric teeth study and morphological analyses. A topographic analysis was performed using the CloudCompare software (https://www.cloudcompare.org, accessed on 24 February 2023). CloudCompare is an open-source software developed for 3D point cloud and mesh processing, alignment, and comparison.

**Figure 3:**
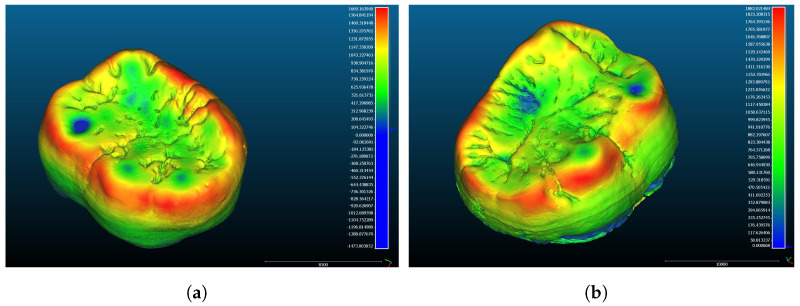
Three-dimensional views of the coronal parts of the teeth with the dental enamel thickness mapped in colour scale. (**a**) Orangutan lower left second molar; (**b**) *Gigantopithecus* lower right second molar.

Whilst the external and internal surfaces of the enamel were separated from each other at the level of the cervical margin, complete surface models of the enamel caps were used for the study. Perforations on the surfaces of the enamel models were initially closed in order to correctly run the automated surface analysis algorithms and topographic mapping (Figure 3), but approaches developed later allowed us to use the software on the perforated models of the worn teeth for orientating and mapping.

**Figure 4:**
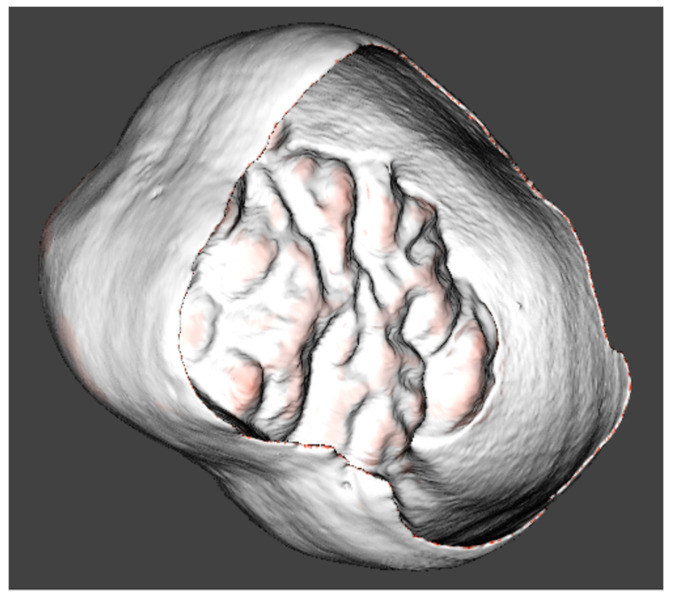
Cervical edge of the enamel cap detected for the 3D model orientation.

Topographic analysis is key to obtaining the overall depiction of the enamel thickness distribution; however, the measurements of the segmented parts of the enamel cap can give more detailed data for comparisons of the results within and between the teeth. The two above-mentioned surfaces share a common borderline at the cervical edge of the enamel cap (Figure 4). This edge contour was found by applying automated algorithms and subsequently used for the preliminary setting of the coordinate system and the orientation of the 3D reconstructions of the studied tooth. A similar technique was proposed for canines [51].

**Figure 5:**
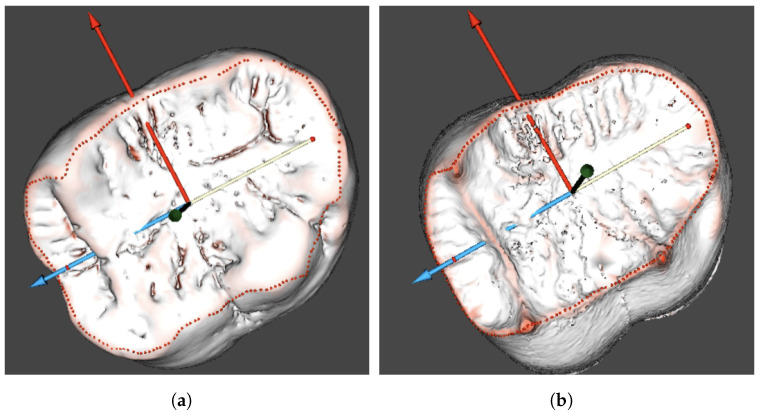
Occlusal surface contour of the *Gigantopithecus* tooth on the enamel (**a**) and dentine (**b**) surfaces. (**a**) Occlusal contour on the enamel; and (**b**) dentine.

Due to multiple irregular pits on the studied teeth, we used a combination of fully automated algorithms that were initially developed for studies on human teeth, and manually operated the procedures. Thus, after the automated surface curvature analysis, the occlusal surface contours on the outer enamel and dentine surfaces were clarified manually, and the orientation was finally set (Figure 5). These contour settings were subsequently used in the segmentation of the measured areas from the enamel cap model. The finalised orientation was used for projecting the borders defined on the outer enamel surface to the dentine surface.

**Figure 6:**
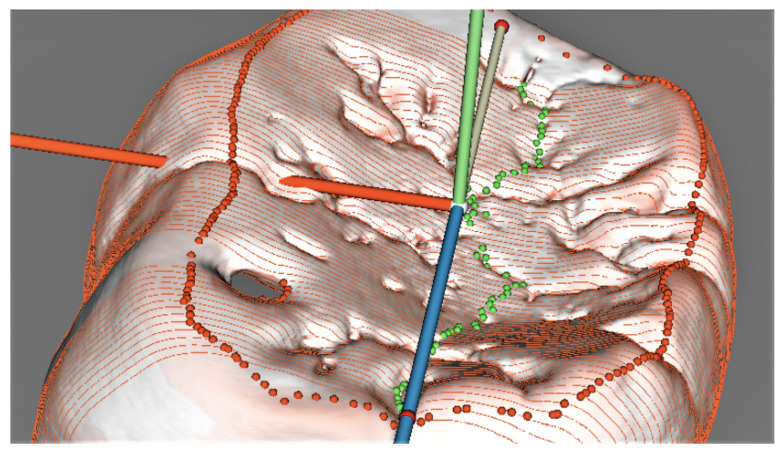
Orangutan tooth with sections on its occlusal surface and the midline set in green points.

The models were subsequently sectioned in the bucco–lingual direction; we obtained 80 sections for each tooth; this number of sections was empirically found to be dense enough to set the midline running in the mesio–distal direction of the tooth crown between its buccal and lingual parts (Figure 6).

**Figure 7:**
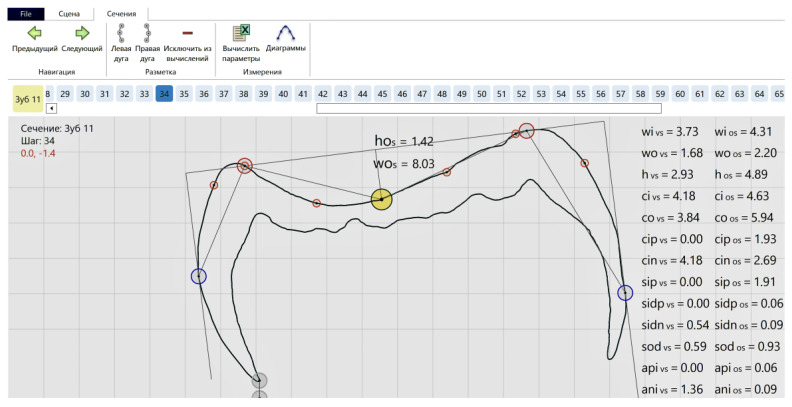
Two-dimensional model of the deepest point (yellow) on the enamel occlusal surface of the orangutan molar.

The midline was set up automatically in the sections (Figure 7), but revisions and manual corrections were necessary in several sections, which ran across pits (especially typical for the orangutan molar) and blocked the correct algorithm automated performance that mislocated the lowermost point position on the contour.

**Figure 8:**
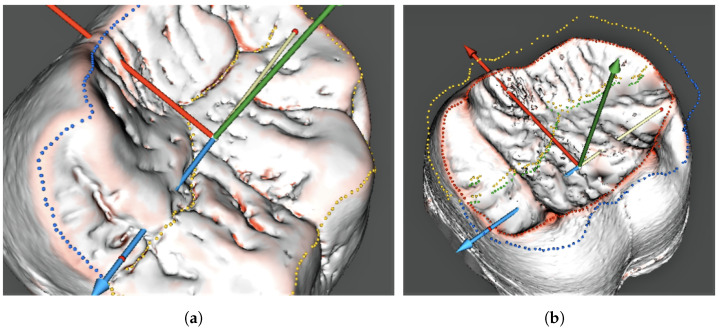
Models of the occlusal surface and midline contours depicting the outer enamel (**a**) and dentine surfaces (**b**). (**a**) Occlusal and midline contours on the enamel; (**b**) occlusal and midline contours on the dentine.

The coordinates of the occlusal surface contour and the midline points set on the outer enamel surface were saved for the subsequent projection onto the dentine surface in the same final orientation (Figure 8). The occlusal surface contour setting on the dentine surface involved a separate procedure; however, the possibility of setting the midline directly on the dentine surface will be studied at later stages due to morphological considerations. The points set along the above-mentioned contours were subsequently used for further virtual segmentation of the inner cusp slopes and to calculate the parameters characterizing the enamel thickness.

Thus, the demarcation described above was performed in both planar and volumetric modes to obtain and compare, in terms of enamel thickness, two distinct parts of the tooth crown. As they are referred to as occlusal surfaces of teeth, named for their buccal and lingual parts, they can also be referred to as the buccal and lingual cusps for their inner slopes. The average enamel thickness parameter was measured for the mentioned sectors. The classical formula for its calculation in 3D mode ($S_{AET3D}$) requires dividing the sector volume ($S_{V}$) by the area of the enamel–dentine junction surface of the corresponding sector ($S_{A}$). However, we propose a modified denominator ($S_{AA}$), which is the average of the enamel–dentine junction surface area ($S_{Aedj}$) and outer enamel surface area ($S_{Aoes}$) of the corresponding sector.

(1) $$\begin{array}{cc} S_{AET3D} & =\frac{S_{V}}{S_{AA}} \end{array}$$

(2) $$\begin{array}{cc} S_{AA} & =\frac{S_{Aedj}+S_{Aoes}}{2} \end{array}$$

**Figure 9:**
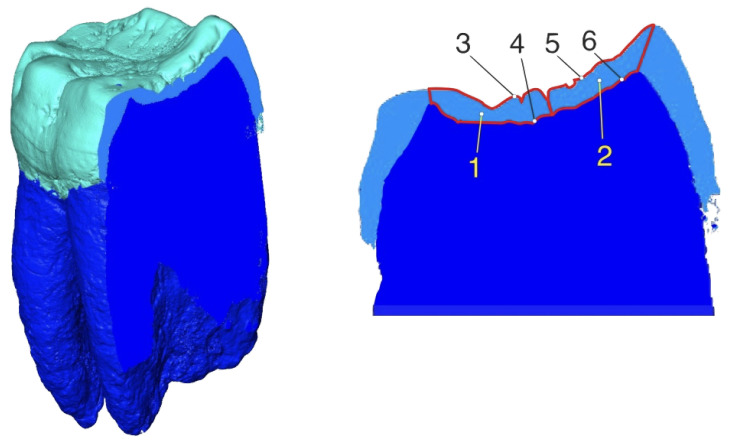
The bucco–lingual section of the *Gigantopithecus*’ lower molar (**left**) with the mapped parameters used for the enamel thickness measurements (**right**). 1—$S_{BA}$—enamel area in the buccal sector; 2—$S_{LA}$—enamel area in the lingual sector; 3—$S_{BCLoes}$—outer enamel surface contour length in the buccal sector; 4—$S_{SBCLedj}$—enamel–dentine junction contour length in the buccal sector; 5—$S_{SLCLoes}$—outer enamel surface contour length in the lingual sector; 6—$S_{SLCLedj}$—enamel–dentine junction contour length in the lingual sector.

Despite the fact that the calculations of the enamel thickness parameters were performed in the 3D mode only, we present an equivalent approach in 2D on a bucco–lingual section of the *Gigantopithecus* tooth (Figure 9) to more clearly explain the enamel thickness measurement concept. The sectioning direction in this particular case has been chosen without following any particular technique, and the sector boundaries do not correspond to those on the studied reconstructions of teeth. This schematic representation of the mapped parameters illustrates them in sectors that are located in two parts of the tooth crown: the buccal and lingual parts. In this planar view, the enamel thickness in a sector will be presented by a corresponding parameter ($S_{AET2D}$). Hence, we have (for the corresponding sector) the area for the numerator ($S_{A}$) and the average of the outer enamel and enamel–dentine junction contours’ lengths ($S_{ACL}$) for the denominator.

(3) $$\begin{array}{cc} S_{AET2D} & =\frac{S_{A}}{S_{ACL}} \end{array}$$

(4) $$\begin{array}{cc} S_{ACL} & =\frac{S_{CLedj}+S_{CLoes}}{2} \end{array}$$

## 3. Results and Discussion

Studies of teeth are an important part of palaeontological research, as new findings shed light on the evolution processes and the distribution of extinct species. One of the most relevant and widely used study techniques is the measuring of enamel thickness, discussed here in terms of methodological approaches, and applied in a test mode to the lower second molars of extinct *Gigantopithecus blacki* and the continental subspecies of orangutan. The proposed tactics are based mainly on previous odontometric studies using image processing and its technological advantages, and new interpretative approaches to morphological and functional issues.

The feasibility of the proposed methods in the current article directly depends on the application of micro-focus tomographic scanning. Thus, imaging and processing techniques have significantly boosted odontological research in different disciplines by providing, among other things, the development and improvement of novel enamel thickness measurement methods. Another important condition for successful studies, in terms of detail and accuracy, is the reconstruction of the morpho-histological layers of teeth. As was shown, a threshold of 40 μm (for voxel edge) is sufficient for accurate measurements of enamel thickness in 3D; the overestimation of the enamel thickness has been observed beyond the mentioned levels [52]. It is worth mentioning that comparisons in imaging techniques have shown that reconstructions obtained both through micro-focus computed tomography and cone-beam computed tomography (CBCT), a conventional 3D imaging method in dental clinical practice, provide a more complete picture of the dental morphology and pathology in comparison with conventional planar radiographic imaging. Nevertheless, CBCT imaging can still be considered an appropriate method for imaging bones rather than teeth.

Though we believe that more accurate and detailed techniques for imaging teeth are also suitable for clinical applications in terms of exposure rate, improving our understanding of the dynamic processes in the masticatory system. Such data could also be extrapolated from palaeontological and palaeoanthropological material that does not actually possess the study potential of teeth from the modern human population. Palaeo-material has fewer limitations when applying different methods of imaging, such as micro-focus tomography, which has become one of the most widely used imaging techniques in enamel thickness studies, due to its non-destructive nature as well as the sizes and histological composition of teeth [6,53,54,55].

We proposed automated algorithms capable of performing calculations of point coordinates and a surface curvature analysis in the presented metric technique, applied to the enamel cap reconstructions in 2D and 3D modes. However, the current study shows that fully automated algorithms developed at earlier stages fail to cope with some specific morphological features, hence requiring further improvements. Nevertheless, for the purpose of this study, semi-automated techniques are applied, in particular for setting the “midline” and demarcating the buccal and lingual parts of the studied teeth. The above-mentioned automated and semi-automated algorithms were applied to reliefs of the enamel, dentine, and cervical enamel edge, but with differences in morphological relevance and methodological meaning. It is worth mentioning that the cervical enamel edge is a curved circular contour that connects the internal and external surfaces of the enamel. This structure is used mainly to orientate the 3D reconstructions of the enamel cap by approximating it to a horizontal plane(s) [20]; original strategies were developed for 2D contours, where the corresponding line served as a starting point for further constructions [7,14]. However, 3D reconstructions provide sufficient conditions for moving beyond the tendency of putting the studied objects on a horizontal plane. For instance, in an orientation according to the enamel edge contour, the “centre of mass” of all of the edge’s point coordinates can be calculated. Such an orientation does not refer to any structures or geometries that are non-relevant to the dental morphology; consequently, each morphological structure or tooth can be oriented according to its own coordinate system.

It is important to mention, however, that the enamel cervical edge is thin and thereby can be easily chipped off on Palaeo-material, which complicates its reliable use in research. In addition, there is no accurate data on the expected stability of this morphological structure throughout the individuals’ lives. Whilst the presented orientation tactic has previously been applied to the occlusal surface, an appropriate approach in the cases of optical scanning techniques applications, in this study the occlusal surface plays another role, which we will discuss below.

The morphological pattern of posterior teeth sectioned in the bucco–lingual direction has traditionally been represented by the contours of two cusps separated by the lowermost depression in the central part of a tooth crown, and hence we use this feature in automated algorithms on 2D contours. In terms of morphology and through a function connection analysis, numerous attempts have been made to classify the buccal and lingual cusps. However, regardless of the terminological differences, there are contradictions in such approaches, as the cusps on both sides of a tooth crown have a common function. For instance, a cusp is classified as “functional” when it comes into contact with the opposing tooth (in a pattern that is supposed to have the meaning of that very function), but surprisingly, it meets a “non-functional” cusp. We will not focus on other attempts of classifying cusps, as they have a very similar type of inconsistency. The latter are supported not only by the functional unity of cusps situated on the buccal and lingual sides, but in morphological terms as well. In this regard, we would like to emphasise the morphological and functional integrative significance of the occlusal surface. However, whilst there is an evident difference between the two sides of a tooth crown, it is difficult to successfully classify them on the level of cusps or cusp slopes, unless we take into consideration the cusp tips and ridges (namely, the mesial and distal ridges of cusps). From this perspective the overlap feature may serve as differentiating criteria. In this case as well, the occlusal surface preserves its interpretative importance as, on the one hand, the aforementioned ridges limit the occlusal surface boundaries, and on the other hand, the occlusal surface is the structure that overlaps (or not) the opposing ridges and tips. Hence, the occlusal surface (and its perimeter) plays a special role in the morphological part of the proposed methodological approaches.

Even though there are examples of studies based on the analysis of occlusal surface contours or cusp tips in 3D reconstructions [20,56], we present measurements that reveal the morphological and functional characteristics of such structures as the occlusal surface and cusps.

The presence of distinct buccally and lingually located cusps in line with their functional inseparability necessitates a discussion on the function itself. Taking the interaction of molars in human dentition as an example, we can skip all stages from the mouth opening until coming into contact of the opposing molars in the lateral occlusion. From here, the mandible starts to move upwards and inwards (usually towards its habitual centric position). In the buccal view, we observe that the outer slopes of the lower molars make contact with the inner slopes on the buccal cusps of the upper teeth. This pattern of masticatory function is usually described as shearing or grinding. It is noteworthy that the morphology and alignment of the molars provides the same pattern of contact on the lingual side, even though this cannot be directly observed naturally. Thus, the outer slopes of the upper lingual cusps guide the inner slopes of the lower lingual cusps. Simultaneously, the cusp slopes, which we have not yet discussed, perform another type of classified function: compressing or stamping. These are both inner slopes, one of which is located on the upper lingual cusp, and the other one is located on the lower buccal. This pair of so-called “stamping” cusps “changes” its function to grinding at the next stage of chewing, when the mandible starts its movement from centric inwards and downwards. This very schematic description of the function distribution pattern may not be seen in a single section (no matter how carefully chosen) or even on a separate tooth. However, it will be observed on one side of a dental arch. The other side of the dental arch will show the opposite direction (from the medial to lateral) of the mandible movement and a different sequence of function changes. We will not overload the article with more detailed descriptions, but it is important to point out that our masticatory systems do not perform a single function at a time. Rather, it is a combination of functions carried out by two interacting dental arches, and these are determined, in many respects, by the morphology of the posterior teeth.

Discussions on the enamel thickness distribution patterns from the positions of dental morphology and function are an important part of many studies. There are already data obtained through the application of a topographic analysis as well as through mapping, and these relate to the occlusal surface and its distribution according to cusps, an analysis from the perspective of “functional” and “non-functional” cusps, and the differences in the enamel thickness on the first and second molars in humans and other species [19,26,29], as well as other relevant issues. Among them, numerous topographic mapping examples related to different species [22] show a similar distribution of the enamel thickness pattern on the buccal and lingual sides of teeth, which can simultaneously be linked with the occlusal interaction of molars on opposing dental arches. Thus, not all cusps or cusp slopes with thick enamel meet similarly thick slopes on opposing teeth. However, one could expect the opposite, especially given the maximum synchronised resistance to functional wear, which is undoubtedly an important adaptive feature. A very similar pattern can be observed on the upper canines [39] that have thicker enamel on their non-contacting labial surfaces. These morphological patterns inherent to separate teeth can be better understood when interpreted from the perspective of the function organisation of the dental arches and process of dental wear. As mentioned above, overlap is one of the most principle features in the alignment of opposing teeth including molars. Pronounced relief of their intact morphology provides sufficient conditions for the inter-cuspation of opposing teeth. However, occlusal surface flattening, which accompanies the process of dental wear, should not result in the loss of overlap, so that the pattern of chewing can be preserved. Therefore, faster wear of the thinner enamel on the inner slopes of the upper buccal and lower lingual cusps prevents molars from sliding out of the mutually overlapping position. Similar observations can be seen on the frontal teeth, when thicker labial enamel preserves the overlap of the upper teeth over the lower teeth. In this context, as shown here, it is especially important to study the morphology of the inner slopes of cusps, which at the same time constitute occlusal surfaces.

The masticatory system develops dynamically. Signs of functional changes can be observed on the inter-proximal surfaces of teeth that gradually wear in parallel with the occlusal surfaces. This results from the functioning of the system’s important feature and the corresponding mechanism that continuously preserves the integrity of the dental arches and contacts between adjacent teeth throughout the individuals’ lives. Teeth move forward in compliance with the inclinations of teeth and the curvatures of dental arches (known as the curve of Spee and the curve of Wilson, respectively). We put special emphasis on the functional and morphological issues in this methodologically oriented work for highlighting the role and importance of dental enamel thickness in the masticatory system as it defines, in parallel with other factors (e.g., eruption time of teeth or inclination of slopes in temporomandibular joints), the essential features of the system development, sustainability, and performance. We believe that this approach may help in interpreting well-known previously observed patterns of enamel thickness distribution. For instance, the thinner enamel on the mesial cusps of both upper and lower first molars can be associated with the formation of temporomandibular joints in children [57], whilst the incisors and canines erupt later to join the mandibular guiding function. It is possible to allow, though hypothetically, for prospects of revealing mechanisms of enamel thickness genetic control in loci that regulate the higher levels of organisation in the masticatory system than the morphology of separate teeth. Another interesting subject for further clinical research is the analysis of enamel thickness distribution pattern effects on malocclusions and after treatment conditions in connection with various factors, such as denture durability, force distribution, or skeletal changes [58].

The issues discussed in the previous paragraphs with respect to the well-known functional aspects of human dentition (largely through clinical applications) can be extrapolated to the masticatory systems of other hominine species as they possess similarities (though dissimilarities are no less important in differentiation), such as thinner enamel on the inner slopes of the upper buccal and lower lingual cusps, which can be observed, as shown, on the teeth of *Pongo*, *Gigantopithecus*, and other primates [29,46,52].

In conformity with the above morphological, functional, and interpretative issues, as well as with previously developed odontometric techniques, we propose this approach to measuring enamel thickness and the analysis of its distribution within the enamel cap volume. Even though a topographic analysis is a very important method for the overall depiction of the enamel thickness distribution, measuring the segmented parts of the enamel cap provides more detailed data for comparisons and analysis within or between the teeth. Therefore, the current work presents the measurements on the occlusal surfaces and the cusp slopes that are the initial stage in the morphologically relevant tactics of mapping the corresponding structures of the studied teeth. The algorithms used have a high level of automation; however, the studied moderately worn lower second molars of *Gigantopithecus* and orangutan require some “manually” operated adjustments.

The measured data are presented as a modified 3D average enamel thickness parameter that deviates from the traditional, ontogenically substantiated uses of the enamel–dentine junction area denominator that depicts the effect of the production of the whole enamel volume through the layer of ameloblasts located at the border between the dentine and enamel. However, we do not deal with intact teeth in our study. Consequently, the objectively measured tissue volume is the result of two natural reverse processes of enamel growth (which stops completely after the formation of the tooth crown) and dental wear. Another factor for proposing the accurate modified parameter is the enamel cap shape (dissimilar with rectangular shapes), which is limited on both sides by two irregular surfaces of enamel and dentine. In addition, the calculation of the outer enamel surface area, both integrated in the denominator parameter or used as a separately calculated parameter, has the potential to be applied in studies concerning morphological changes appearing as a result of dental wear. The complete layout of the parameters measured and calculated in this study for the lower second molars of *Gigantopithecus* and orangutan are presented in Table 1.

The data show that both teeth have thinner enamel on the inner slopes of their lingual cusps. This corresponds with the expected result, as similar observations on the enamel thickness distribution can be seen while visually studying topographic maps of other primates’ teeth. The *Gigantopithecus* tooth has thicker enamel on the occlusal surface in comparison with the similarly worn tooth of the orangutan. The small sample does not provide the opportunity to detect any clear differentiating features for these species. Therefore, we plan to expand the sample in further studies including other teeth of extant and extinct primates that were found at the same locations as the presented teeth. Another methodological issue to be discussed in the future refers to the sizes of teeth, and we will conduct combined odontometric and enamel thickness studies.

We plan to focus on the methodological approaches that were applied and tested in this study in order to overcome the technique’s limitations. Further improvements will be proposed for orientation algorithms that will support the tracking of changes in studies on the dynamic processes of the masticatory system. Measurements of the enamel thickness on the outer cuspal slopes are of interest; therefore, corresponding algorithms should be developed. In general, advances in digital dental metric techniques should be expected in the improvements of automatically running algorithms for the development of accurate, replicable, and morphologically relevant measurement methods that cover a wider variety of samples in flexible, adjustable, and multi-task systems with integrated analytical functions. Such tasks can be effectively resolved using current technology.

## 4. Conclusions

New methodological approaches to enamel thickness measurements were proposed in this article. They are based on the interpretations of dental morphology and function and provide improvements in research tactics, taking advantage of digital imaging techniques and image analysis. The proposed tactics have the potential for further improvements, accounting for the morphological considerations characteristic to the different species. In particular, the methodological part was performed using automated algorithms for the orientation, mapping, and segmentation of the tooth enamel volumetric reconstruction. The methodological approaches were tested on the lower molars of *Gigantopithecus* and orangutan, and we plan to use these methods on a wider sample in the near future.

## Figures and Tables

**Table 1:** Enamel area, volume and thickness data, and analysis.

	*Gigantopithecus*	Orangutan	Percentage of Segment	
			***Gigantopithecus***	**Orangutan**
Outer enamel surface area, mm${}^{2}$				
Occlusal surface	249.96	160.62	100	
Inner slope of buccal cusp	137.22	92.73	54.90	57.73
Inner slope of lingual cusp	112.74	67.89	45.10	42.27
Enamel–dentine junction area, mm${}^{2}$				
Occlusal surface	227.46	142.95	100	
Inner slope of buccal cusp	122.61	83.53	53.90	58.43
Inner slope of lingual cusp	104.85	59.42	46.10	41.57
Enamel volume, mm${}^{3}$				
Occlusal surface	170.60	95.78	100	
Inner slope of buccal cusp	93.18	56.80	54.62	59.31
Inner slope of lingual cusp	77.59	39.22	45.48	40.95
Three-dimensional average enamel thickness, mm${}^{3}$				
Occlusal surface	0.751	0.673	100	
Inner slope of buccal cusp	0.764	0.681	101.73	101.19
Inner slope of lingual cusp	0.735	0.657	97.87	97.62

## Data Availability

The data presented in this study is available on request from the corresponding author. The data is not publicly available due to the policy of the owner.

## Abbreviations

The following abbreviations are used in this manuscript:

ADO	Automated digital odometry
CBCT	Cone-beam-computed tomography
CT	Computed tomography
μCT	Micro-computed tomography
TIN	Triangulated irregular network
